# Supplementary material for: Exploring the relationship between job characteristics and infection: Application of a COVID-19 job exposure matrix to SARS-CoV-2 infection data in the United Kingdom
Source: Scand J Work Environ Health. 2023 Mar 30;49(3):171–81. doi: 10.5271/sjweh.4076 (PMC10621898; doi:10.5271/sjweh.4076)

Exploring the relationship between job characteristics and infection: Application of a COVID-19 job exposure matrix to SARS-CoV-2 infection data in the United Kingdom[1]

by Sarah Rhodes, PhD,[2] Sarah Beale, MSc, Jack Wilkinson, PhD, Karin van Veldhoven, PhD, Ioannis Basinas, PhD, William Mueller, MSc, Karen Marieke Oude Hengel, PhD, Alex Burdorf, PhD, Susan Peters, PhD, Zara A Stokholm, PhD, Vivi Schlünssen, PhD, Henrik Kolstad, PhD, Anjoeka Pronk, PhD, Neil Pearce, PhD, Andrew Hayward, MD, Martie van Tongeren, PhD

1. *Supplementary Material*
2. *Correspondence to: Division of Population Health, Health Services Research & Primary Care, University of Manchester, Manchester, UK. [E-mail: sarah.a.rhodes@manchester.ac.uk]*

Table S1. Odds ratios (95% CIs) by level of exposure for Domains 1-4 of the Job Exposure Matrix for two cohorts (CIS and Virus Watch). Models adjusted for age quintile, sex, ethnicity, IMD, region, household size, urban vs rural area, and presence of health conditions. N = 244,470 and N=16,479 for CIS and Virus Watch cohorts respectively. See table 1 for domain descriptions.

	Domain 1 (Number)		Domain 2 (Nature)		Domain 3 (Surfaces)		Domain 4 (Location)	
	CIS	Virus Watch	CIS	Virus Watch	CIS	Virus Watch	CIS	Virus Watch
No Risk	1	1	1	1	1	1	1	1
Low Risk	1.07 (1.02-1.12)	0.99 (0.88-1.11)	1.10 (1.06-1.15)	1.13 (1.02-1.25)	1.13 (1.08-1.19)	1.19 (1.07-1.32)	1.14 (1.04-1.24)	1.08 (0.84-1.37)
Elevated Risk	1.11 (1.07-1.16)	1.22 (1.11-1.35)	1.17 (1.13. 1.22)	1.17 (1.07-1.27)	1.08 (1.03-1.14)	1.17 (1.04-1.30)	1.20 (1.12-1.27)	1.03 (0.87-1.22)

	Domain 1 (Number)		Domain 2 (Nature)		Domain 3 (Surfaces)		Domain 4 (Location)	
	CIS	Virus Watch	CIS	Virus Watch	CIS	Virus Watch	CIS	Virus Watch
High Risk	1.19 (1.14-1.24)	1.24 (1.12-1.37)	0.97 (0.90-1.04)	1.48 (1.26-1.72)	1.15 (1.11-1.20)	1.26 (1.15-1.38)	1.12 (1.08-1.16)	1.19 (1.10-1.29)
Missing	1.18 (1.14-1.23)	0.89 (0.70-1.11)	1.18 (1.14-1.23)	0.89 (0.71-1.12)	1.17 (1.13-1.22)	0.90 (0.72-1.13)	1.18 (1.14-1.23)	0.89 (0.70-1.11)

	Domain 5 (Distancing)		Domain 6 (Face covering)		Domain 7 (Insecurity)		Domain 8 (Migrants)	
	CIS	Virus Watch	CIS	Virus Watch	CIS	Virus Watch	CIS	Virus Watch
No Risk	1	1	1	1	1	1.10 (0.35-2.86)	1	1
Low Risk	1.06 (1.02-1.10)	1.04 (0.95-1.14)	1.07 (1.02-1.12)	1.29 (1.16-1.44)	0.83 (0.55-1.26)	1	0.98 (0.95-1.01)	1.00 (0.93-1.07)
Elevated Risk	1.15 (1.11-1.21)	1.22 (1.11-1.35)	1.15 (1.11-1.20)	1.12 (1.03-1.22)	0.80 (0.53-1.21	1.07 (0.99-1.15)	1.09 (0.99-1.19)	1.56 (1.23-1.96)
High Risk	1.28 (1.22-1.35)	1.54 (1.36-1.74)	1.05 (0.90-1.23	1.47 (1.11-1.93)	1.00 (0.63-1.58)	1.38 (0.76-2.40)	1.42 (1.19-1.69)	0.93 (0.52-1.59)
Missing	1.18 (1.14-1.23)	0.89 (0.70-1.11)	1.18 (1.14-1.23)	0.89 (0.70-1.11)	0.89 (0.59-1.35)	0.83 (0.66-1.03)	1.08 (1.05-1.12)	0.81 (0.64-1.01)

Table S2. Spearman's rank correlation matrix for 8 domains of the JEM, using first available JEM code.
N=176 713 participants with available SOC in the CIS. See table 1 for domain descriptions.

	Number	Nature	Surfaces	Location	Distancing	Face-covering	Insecurity	Migrant
Number	1							
Nature	0.8412	1						
Surfaces	0.8273	0.9026	1					
Location	0.8116	0.8397	0.7992	1				
Distancing	0.8251	0.8359	0.7967	0.7821	1			
Face-covering	0.6681	0.6304	0.5483	0.7725	0.6533	1		
Insecurity	0.3025-	0.2946	0.314	0.2698	0.3467	0.1579	1	
Migrants	0.0555	0.0219	0.0252	0.0025	0.0492	0.0086	0.2042	1

Figure S1. Odds ratios for COVID-19 by level of exposure for 6 domains of the Job Exposure Matrix from Virus Watch, adjusted for participants' vaccination status in addition to adjustment provided in main analyses. As above, please see Table 1 for explanation of abbreviations and domain descriptions. Domains relating to insecurity and migrant workers not displayed here due to imprecision.

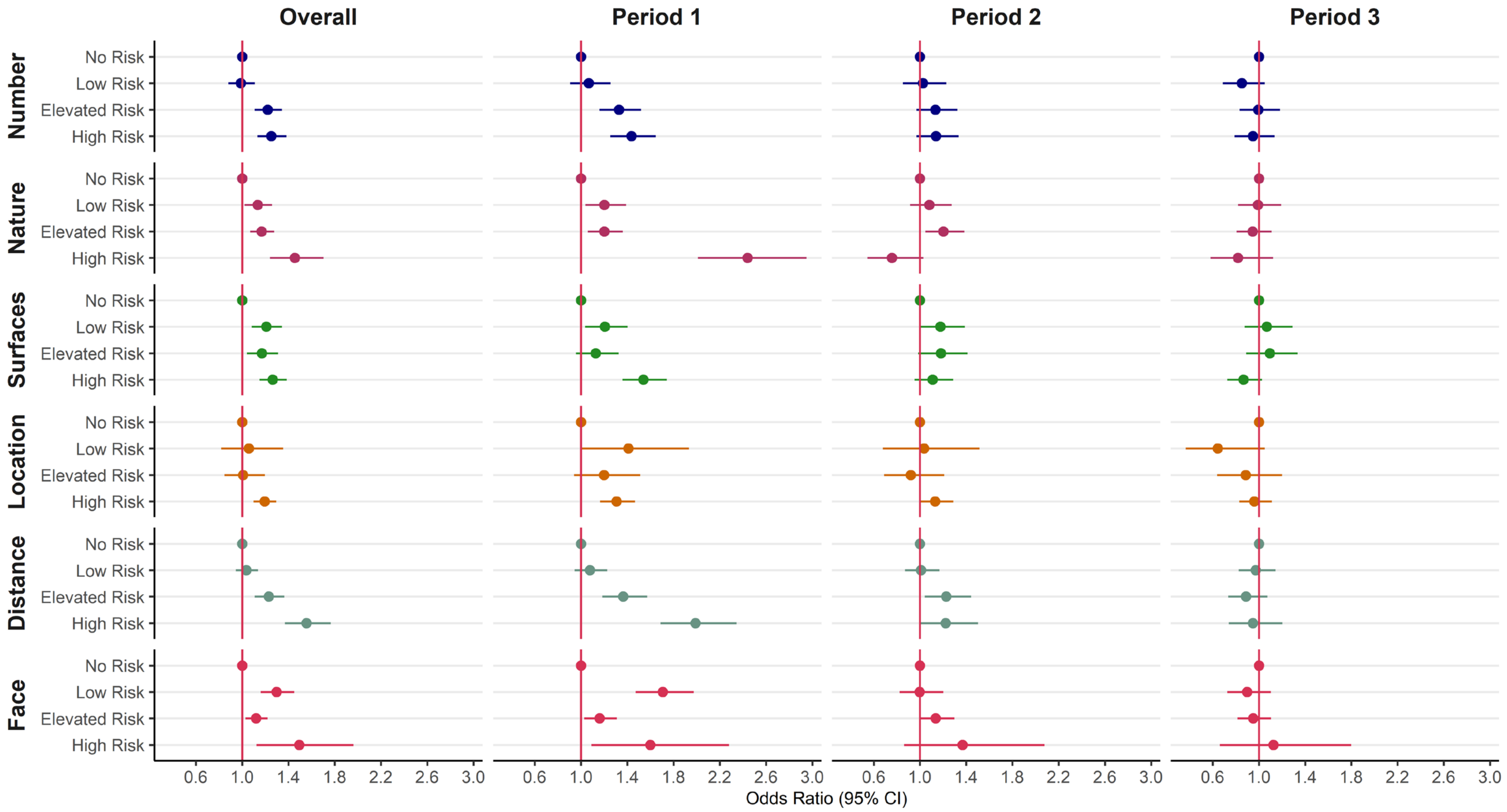

Supplement: Supplementary material [file SJWEH-49-171-S001.pdf]
